# Health Literacy, Self-Efficacy, and Associated Factors Among Patients with Diabetes

**DOI:** 10.3928/24748307-20180313-01

**Published:** 2018-04-12

**Authors:** Xin Yi Xu, Angela Yee Man Leung, Pui Hing Chau

## Abstract

**Background:**

High levels of health literacy (HL) and self-efficacy (SE) are important steps in managing diabetes. Previous studies have investigated the role played by the individual constructs (HL or SE) on self-care behaviors and health outcomes in patients with diabetes. However, our understanding of the relationship between HL and SE is limited.

**Methods:**

Literature was searched in PubMed, Medline (via OvidSP), CINAHL (via EBSCOhost), ProQuest Medical Library, and Science Direct using keywords “diabetes,” “diabetic,” “DM,” “T1DM,” “T2DM,” “health literacy,” “HL,” “common HL,” “diabetes HL,” “SE,” “general SE,” and “diabetes SE.” The keywords were limited by “MeSH terms” and “Title and Abstracts.”

**Key Results:**

Eleven studies were included in this systematic review. Communicative and critical HL were prominent in this relationship. Among the 11 reviewed studies, a positive relationship between communicative/critical HL and SE in diabetes care was illustrated, but the relationship between functional HL and SE remained controversial. Factors positively associated with HL and SE were educational level, employment status, annual income, social support, clarity of the physician's explanation, and empowerment perception.

**Discussion:**

Health professionals should act to improve communicative and critical HL so that patients may be more confident in managing diabetes. Clarity in health professionals' explanations and social support would be helpful in enabling patients with diabetes to build up their SE and HL. **[*HLRP: Health Literacy Research and Practice*. 2018;2(2):e67–e77.]**

**Plain Language Summary:**

This study is the first systematic review to investigate the relationship between health literacy (HL) and self-efficacy (SE) among persons with diabetes. Even though the relationship between HL and SE needs to be further explored, communicative and critical HL were found to be positively associated with SE. Therefore, to support people in building up SE, health professionals should consider actions that support communication and critical thinking in health settings.

The prevalence of diabetes has been increasing in the last few decades (Chen, Magliano, & Zimmet, 2011; Danaei et al., 2011; Whiting, Guariguata, Weil, & Shaw, 2011). This chronic illness has gradually become the leading cause of morbidity and mortality worldwide (Lozano et al., 2012). People with type 1 or type 2 diabetes mellitus have an increased risk of developing cardiovascular diseases (American Diabetes Association, 2013) and other long-term complications of diabetes such as damage to eyes, kidneys, and nerves (Icks et al., 2009; Min, Stephens, Kumar, & Chudleigh, 2012).

People with diabetes need self-care to prevent complications and improve their quality of life (American Diabetes Association, 2010). Diabetes self-care behaviors refer to activities such as following a healthy diet, doing physical activity regularly, adhering to medications, and controlling blood glucose (Shrivastava, Shrivastava, & Ramasamy, 2013). People with diabetes who have self-care behaviors will have good glycemic control and improvement in quality of life (Odegard & Capoccia, 2007; Povey & Clark-Carter, 2007). Many patients could reduce the chances of developing long-term complications by adhering to self-care behaviors.

One factor that has a great influence on diabetes self-care is health literacy (HL). HL refers to “the cognitive and social skills which determine the motivation and ability of individuals to gain access to, understand and use information in ways which promote and maintain good health” (Nutbeam, 1998; Nutbeam, 2000). Based on this definition, HL is not only a personal resource leading to personal benefits, but also an attribute contributing to social benefits (due to effective communication between all parties in the community) and supporting the development of social and political actions, as well as individual actions (Nutbeam, 2000); therefore, HL is divided into three subdomains: functional HL, communicative (or interactive) HL, and critical HL (Nutbeam, 2000). We are now aware that most HL studies focus on the investigation of functional HL. However, some studies, such as Y. J. Lee et al. (2016), E.H. Lee, Y.W. Lee, and Moon (2016), Reisi et al. (2016), and Inoue, Takahashi, and Kai (2013), have used conceptual frameworks with three subdomains of HL to assess self-care issues among patients with diabetes. In addition, a Japanese team of researchers (Ishikawa, Takeuchi, & Yano, 2008) developed a measuring tool to access HL, showing three subdomains of HL among Japanese patients with diabetes.

HL has been proposed as an important capacity that people should possess to maintain good health (Berkman et al., 2011). Many studies have shown the positive relationship between HL and diabetes self-care (Al Sayah, Majumdar, Williams, Robertson, & Johnson, 2013; Bohanny et al., 2013; Osborn, Cavanaugh, Wallston, & Rothman, 2010). Patients with inadequate HL have less capacity to perform self-care behaviors than their more health-literate counterparts, eventually leading to poor clinical outcomes and higher morbidity and mortality (Baker, Wolf, Feinglass, & Thompson, 2008). For example, patients with inadequate HL were less likely to engage in physical activity, which constitutes a vital part of self-care behaviors, and thus were more likely to have poor glycemic control (E. H. Lee et al., 2016; van der Heide et al., 2014). Inadequate HL was considered an obstacle to absorbing essential health information in diabetes care (Bains & Egede, 2011; Sarkar, Fisher, & Schillinger, 2006). In view of the relationship between HL and diabetes self-care, interventions were proposed and implemented to alleviate the effect of inadequate HL on self-care in diabetes.

Another factor known to be associated with diabetes self-care is self-efficacy (SE) (Bohanny et al., 2013). In a recent study, SE contributed to a significant explanation of the variance in self-care behaviors (Bohanny et al., 2013). SE is one of the fundamental concepts of Social Cognitive Theory. It refers to “the belief in one's capacity to organize and execute the courses of action required to manage a prospective situation” (Bandura, 1995). How people will perform in the future depends largely on whether they believe they have the capacity to act (Bandura, 1997). SE is a central determinant that directly affects health behaviors (Bandura, 2004). Patients with higher SE are more likely to have better adherence to self-care tasks (Reisi et al., 2016). Both general SE and disease-specific SE may affect patients with diabetes self-care behavior (King et al., 2010; Trief, Teresi, Eimicke, Shea, & Weinstock, 2009). High SE is needed for better diabetes self-management.

Both HL and SE are vital constructs in diabetes care, supporting improvements in quality of life and reducing complications. Without behavioral capability (knowledge and/or skills) in performing a specific act like managing blood glucose, SE alone is not able to generate good diabetes self-care behavior (Bandura, 2001). The interaction between HL and SE may positively predict good health care behavior. Previous studies (Al Sayah et al., 2013; Bohanny et al., 2013; King et al., 2010; Reisi et al., 2016) have investigated the role played by the individual constructs (HL or SE) on self-care behaviors and health outcomes in patients with diabetes. However, our understanding of the relationship between HL and SE is limited. If a relationship is established, interventions in diabetes care should focus on both HL and SE, rather than on a single construct.

HL can be considered as three subdomains: (1) functional HL is the basic reading and writing skills that people need to possess to function effectively in everyday life; (2) communicative HL is the advanced skill needed for a person to extract useful information in the communication process; and (3) critical HL refers to the more advanced skills for thinking about information critically and applying it to manage daily life (Nutbeam, 2000). This article investigates the relationship between HL (as a whole) and SE, and the relationships between the different subdomains of HL and SE.

## Methods

### Search Strategy

Specific keywords, MeSH terms, subheadings, and index terms were used in the search. Electronic databases searched included PubMed, Medline (via OvidSP), CINAHL (via EBSCOhost), ProQuest Medical Library, and Science Direct. We limited the search to studies published in English. The search was done from April to May 2017.

The first line aimed to establish the characteristics of the population, with keywords “diabetes,” “diabetic,” “DM,” “T1DM,” and “T2DM.” The second line aimed to find different aspects of health literacy, with keywords “health literacy,” “HL,” “general health literacy,” and “diabetes health literacy.” The third line aimed to find a comprehensive definition of self-efficacy, with keywords “self-efficacy,” “general self-efficacy,” and “diabetes self-efficacy.” Synonyms in the same line were combined with “or,” and the key lines were combined with “and.” They were then used to search for evidence in the databases.

During the search process, we browsed the titles and abstracts of the studies first. If the study was related to the review topics, then the full text was extracted, and the details of the study read.

### Inclusion and Exclusion Criteria

This review included studies of the relationship between HL and SE with participants who had type 1 or type 2 diabetes, published in the last 20 years (April 1997 to April 2017). The exclusion criteria were not being a primary study and written in a language other than English.

### Data Extraction and Synthesis

Data were extracted under a structured form. One reviewer extracted data from each study, whereas the others read each article to check the accuracy and completeness of the extracted data. The data extracted from the reviewed studies included type of studies, number of participants, participants' characteristics, study methods, measurements of study outcomes, results, and conclusion. Through the review process, a retrieval rule was followed, which implied the integrity of the selection process in the study.

### Critical Appraisal of the Reviewed Studies

The quality of the reviewed studies was assessed by a standardized critical appraisal instrument from the Study Quality Assessment Tools offered by the National Institutes of Health (National Institutes of Health, 2014). Two reviewers assessed the quality of the reviewed studies separately under this standardized critical appraisal instrument. Discrepancies were solved by the third researcher. The study quality was assessed by the following criteria: a clear research question, an appropriate research method, an explicit description of study sampling, data collection and data analysis, a proper use of measurements, good consideration of ethical issues, and potential bias (National Institutes of Health, 2014). According to the criteria, each study was assigned an overall quality rating: good, fair, and poor. This classification was based on the number of criteria the study met and the risk for potential bias. After a critical appraisal process completed by two independent reviewers, only one study (McCleary-Jones, 2011) was deemed to be low quality because this study used an insufficient number of participants.

## Results

### Study Characteristics

**Figure 1.**
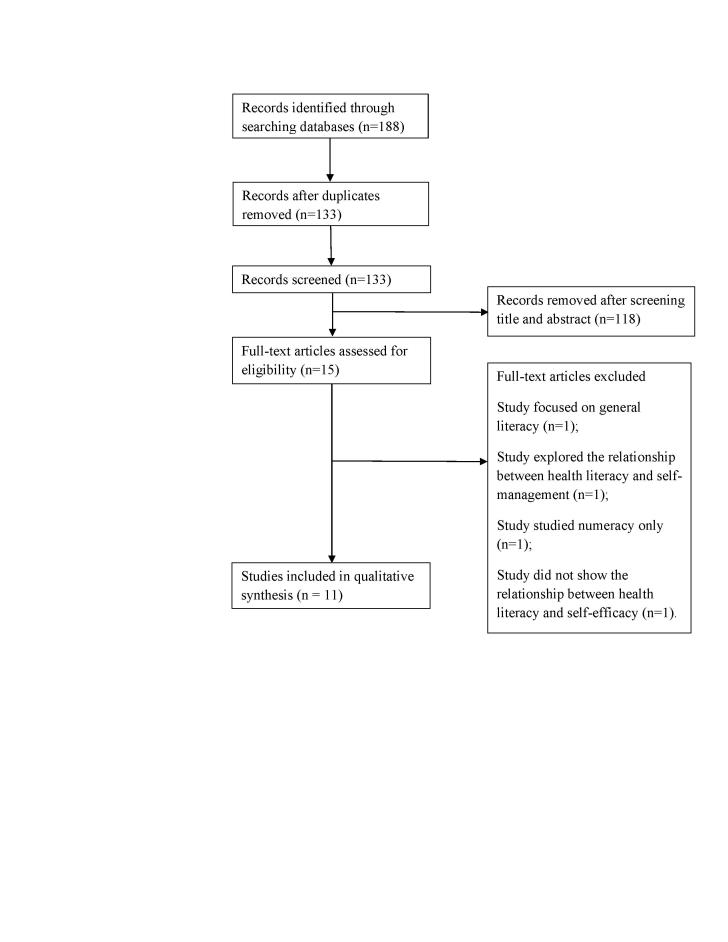
Flowchart showing data selection process.

**Figure 1** shows the results of the search. A total of 188 studies were identified. We excluded 173 articles that were duplicated or met the exclusion criteria. We then excluded four more articles because one studied general literacy (not HL), one explored the relationship between SE and self-management, another focused on numeracy instead of HL, and the last one did not show the relationship between HL and SE. A total of 11 articles were included in this review.

A total of 3,471 participants were included in these reviewed papers. Participants were all adults with a range of geographical locations, nationalities, ethnicities, ages, and educational status. Clinics, health centers, community centers and hospitals were used to conduct surveys. **Table 1** shows the evidence of the reviewed studies.

There were various kinds of measurements of HL. Two studies (Bohanny et al., 2013; White, Osborn, Gebretsadik, Kripalani, & Rothman, 2013) used the Short Test of Functional Health Literacy in Adults (S-TOFHLA) scale to assess participants' functional HL. Another two studies (McCleary-Jones, 2011; Osborn et al., 2010) used the Rapid Estimate of Adult Literacy in Medicine (REALM) to assess functional HL (Hoffman-Goetz, Donelle, & Ahmed, 2014). A scale that was developed in Japan to assess the functional, communicative, and critical HL of patients with diabetes was applied in four studies (Inoue et al., 2013; E. H. Lee et al., 2016; Y. J. Lee et al., 2016; Reisi et al., 2016). The other studies applied different HL scales to measure HL among their participants.

Measurements of SE also varied. The Perceived Diabetes Self-Management Scale was used in three studies (Al Sayah, Majumdar, Egede, & Johnson, 2015; Osborn et al., 2010; White et al., 2013). The 4-item Self-Care Ability in Diabetes Care Profile was used in two studies (Inoue et al., 2013; Ishikawa & Yano, 2011). The Diabetes Self-Efficacy Scale, the 14-item Chinese version Self-Efficacy for Diabetes Management Scale, and the Diabetes Management Self-Efficacy Scale were used by five studies (Bohanny et al., 2013; E. H. Lee et al., 2016; Y. J. Lee et al., 2016; McCleary-Jones, 2011; Reisi et al., 2016) to assess patients' SE. The other study applied the Stanford SE questionnaire to measure SE among its participants (Zuercher, Diatta, Burnand, & Peytremann-Bridevaux, 2017).

## Relationship Between Health Literacy and Self-Efficacy

**Figure 2.**
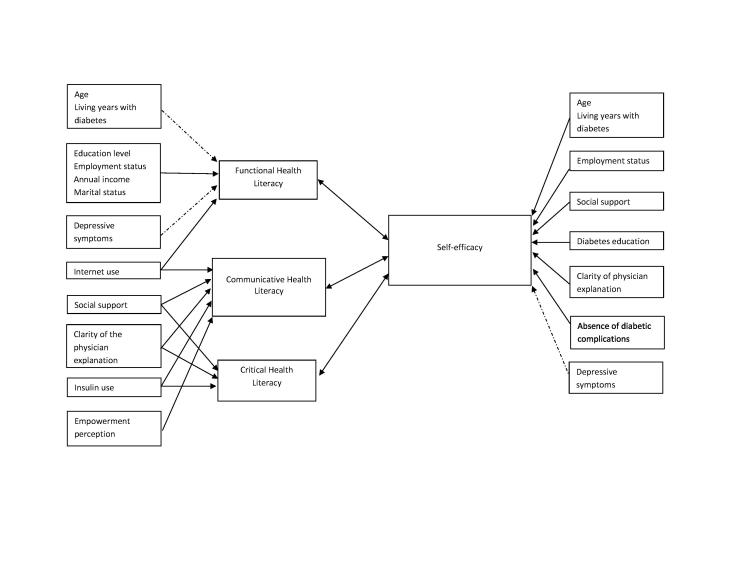
The relationship between health literacy and self-efficacy, and their associated factors. Solid arrow, postive relationship. Dotted arrow, negative relationship.

**Figure 2** shows the relationship between HL and SE. Among the 11 reviewed studies, positive relationships between HL and SE were found in eight (Bohanny et al., 2013; Inoue et al., 2013; Ishikawa & Yano, 2011; E. H. Lee et al., 2016; Y. J. Lee et al., 2016; Osborn et al., 2010; Reisi et al., 2016; Zuercher et al., 2017). Splitting HL into three subdomains (functional HL, communicative HL, and critical HL), 10 studies assessed the relationship between functional HL and SE. The cross-sectional study conducted by Osborn et al. (2010) showed that functional HL had a direct association with SE. The study by Bohanny et al. (2013) showed similar findings but emphasized that functional HL explained a greater percentage of variance in SE than diabetes education and employment status. In the study conducted by Zuercher et al. (2017), which only explored functional HL, the researchers concluded that the SE score was significantly lower for patients with low functional HL than for those with high functional HL. However, another three studies yielded different findings and showed that SE and HL has no relationship (Al Sayah et al., 2015; McCleary-Jones, 2011; White et al., 2013).

In the study by Inoue et al. (2013), communicative and critical HL were positively associated with SE for diabetes management, but functional HL was not. The study by Y. J. Lee et al. (2016), similar to that of Inoue et al. (2013), found that critical and communicative HL accounted for more variance of the relationship with SE than functional HL did. Reisi et al. (2016) showed that all communicative, functional, and critical HL was positively related to SE after adjusting for potential confounders. A similar result was also found in the E. H. Lee et al. (2016) study, in which all communicative, functional, and critical HL was positively related to SE. Ishikawa and Yano (2011), on the other hand, concluded that communicative HL had a positive correlation with SE in diabetic self-care.

## Factors Affecting Health Literacy and Self-Efficacy

**Figure 2** also summarized the factors associated with HL and SE from the reviewed studies. Five factors (educational level, employment status, annual income, marital status, and Internet use) were positively associated with functional HL, although three factors (age, years of living with diabetes, and depressive symptoms) had a negative association. This implied that those who had higher educational level, had higher annual income, were currently employed, were married, and used the Internet frequently would have higher functional HL. By contrast, those who were older, had lived more years with diabetes, or had depressive symptoms were more likely to have inadequate functional HL than their counterparts. On the other hand, five factors (Internet use, social support, clarity of the physician's explanation, insulin use, and empowerment perception) were positively associated with communicative HL. This implies that those who used the Internet more, had more social support, received clear explanations from physicians, used insulin, and perceived themselves as being empowered were more likely to have better communicative HL. Another four factors (social support, clarity of the physician's explanation, insulin use, and empowerment perception) were positively associated with critical HL. This implied that those who had more social support, received clear explanations from physicians, and used insulin were more likely to have higher critical HL.

On the other hand, seven factors (age, years of living with diabetes, employment status, social support, diabetes education, clarity of physician's explanation, and absence of diabetic complications) were associated with SE. Only depressive symptoms were negatively associated with SE.

Three factors (employment status, social support, and clarity of the physician's explanation) were identified as common factors having positive relationships with both HL and SE, although depressive symptoms were the only common factor that had negative relationships with both HL and SE.

## Discussion

The result of this systematic review revealed the relationships between HL and SE among 3,471 patients with diabetes of various ethnicities and nationalities. Positive relationships were established between communicative HL and SE, and between critical HL and SE. However, inconsistent findings were noted in the relationship between functional HL and SE, with some studies reporting a positive relationship between HL and SE and others showing no relationship between HL and SE (Al Sayah et al., 2015; Bohanny et al., 2013; E. H. Lee et al., 2016; Y. J. Lee et al., 2016; McCleary-Jones, 2011).

One of the possible reasons for the controversial relationship between functional HL and SE is the use of a HL tool that basically measures functional HL (such as the REALM or the S-TOFHLA). When functional HL and SE were considered, the relationship was inconsistent. However, when communicative HL/critical HL were considered, their relationships with SE were positive. Such findings are further supported by two other studies (Ishikawa & Yano, 2011; Y. J. Lee et al., 2016). Therefore, inconsistent findings in the functional HL-SE relationship could be related to the use of inconsistent measures. By 2013, more than 51 HL measures had been developed in different countries (Haun, Valerio, McCormack, Sorensen, & Paasche-Orlow, 2014). It is recommended that researchers select an appropriate HL measure according to the aims of individual studies.

The difference between measures of HL that are objective tests and subjective tests should be considered as one of the reasons for the inconsistency of the results in the reviewed studies. For example, REALM (Murphy, Davis, Long, Jackson, & Decker, 1993) and TOFHLA (Parker, Baker, Williams, & Nurss, 1995) were the objective HL measures, whereas the scale developed in Japan (Ishikawa, Takeuchi, & Yano, 2008) was the subjective HL measure. A complex interaction between self-reported (subjective) measures of HL and SE may exist. People with a high level of SE may overestimate their own abilities to understand and interpret health information, and therefore may subjectively rate their HL high. Consequently, the self-reported HL level is high. Further, one of the studies that reports no relationship between functional HL and SE is of low quality, so the result may not be that reliable (McCleary-Jones, 2011).

Another possible reason for the inconsistent relationship may be the different study populations. One of the inclusion criteria of our review was that participants in the study should have type 1 or type 2 diabetes. However, most of the patients with type 1 diabetes had been diagnosed in childhood. This may have affected their HL and SE compared to the patients with type 2 diabetes (American Diabetes Association, 2012). Four of the available studies included patients with both type 1 and type 2 diabetes (McCleary-Jones, 2011; Osborn et al., 2010; White et al., 2013; Zuercher et al., 2017). Two of them showed that there was no relationship between HL and SE (McCleary-Jones, 2011; White et al., 2013).

The two reviewed studies used multifaceted HL scales to investigate the relationships between the individual subdomains of HL (functional, communicative, and critical HL) and SE (Inoue et al., 2013; Y. J. Lee et al., 2016). The results showed that communicative and critical HL explained more variance in SE than functional HL. This finding implies that people who possess functional HL might not have enough SE in diabetes care. Until people possess both communicative and critical HL, they may not perceive sufficient confidence in their self-care abilities in diabetes (i.e., they may have low SE). Therefore, to support people in building up SE, health professionals should consider actions that support communication and critical thinking in health settings. Communicative HL refers to enhanced skills that people should possess to manage their health in collaboration with clinical practitioners, whereas critical HL refers to the capacity to analyze health information critically and to develop actions to address possible barriers in health decisions (Nutbeam, 1999). These capacities can only be exercised when the health care setting is open to discussion and negotiation. As shown in this review, the clarity of the physician's explanation is a factor determining HL and SE. People can build up their HL and SE when physicians and other health professionals explain the treatment options clearly.

This review also showed that social support was a factor associated with communicative HL, critical HL, and SE. Therefore, in addition to clinical practitioners, relatives, friends, and peers who provide social support to patients with diabetes can help to build up their HL and SE. By contrast, depressive symptoms were found to be a factor negatively associated with both functional HL and SE. This implies that patients with depressive symptoms might not have enough HL and SE and would need more attention from clinical practitioners or support in decision-making regarding their diabetes care. Because patients with diabetes who experience depressive symptoms have poorer physical and mental function that incurs higher health care costs (Arshad & Alvi, 2016), support given to these patients could result in cost savings.

There is increasing research studying the relationship between HL and diabetes care, as well as that between SE and diabetes care. Positive relationships between HL and diabetes self-care, or between SE and diabetes self-care are well illustrated (Al Sayah et al., 2013; Bohanny et al., 2013; Walker, Smalls, Hernandez-Tejada, Campbell, & Egede, 2014). The current review study contributed to our understanding of the relationship between HL and SE in patients with diabetes. The current findings remind us to address the relationship between particular subdomains of HL (communicative HL and critical HL) and SE in diabetes care. Although previous interventional studies have done well in advocating the importance of functional HL in diabetes care, it is time for us to think about the possible contribution that could be made by communicative HL and critical HL in diabetes care.

When health professionals encourage patients to embrace self-care behaviors, interactions between health professionals and patients should also be encouraged. Health professionals should be ready to receive questions from patients with diabetes in terms of treatment choices or alternatives in conducting self-care. Health professionals should make an effort to offer diabetes-related health education in a clear and systematic way, and be open in their discussions with patients (Kripalani et al., 2010). Patients may have difficulty understanding medical terminology and health instruments (Davis, Crouch, Wills, Miller, & Abdehou, 1990), which are common in communication. Such difficulties can prevent patients from receiving self-care knowledge from clinical practitioners (Coleman & Berenson, 2004). Feeling connected to health professionals (including nurses) allows patients to acquire more health information and gain confidence in exchanging their knowledge and experience (Bhandari & Kim, 2016). Only working with patients in this way can increase patients' SE in diabetes care. Identifying factors that are positively or negatively associated with HL or SE could provide insights to health professionals, helping them to understand that patients with advanced age, with less education, or with depressive symptoms are more likely to have low HL or low SE. Based on this finding, health professionals could pay more attention to this group of patients and support them in developing the capacity to communicate with health professionals or make enquiries, if any, in diabetes care.

This review also has several limitations. First, only primary studies written in English were included in this review. Studies written in other languages, such as Chinese, were omitted; therefore, situations in non–English-speaking countries may not be completely represented in this review. Second, we have not investigated the mediating or moderating effects of HL on the relationship between SE and self-care. Further investigation could be made in this area in the future. Third, because only cross-sectional studies were included in this review, we are uncertain about the causal relationship between these two constructs. Longitudinal studies could be reviewed to confirm the causal relationship between HL and SE among patients with diabetes. Future research can also look for mediators or moderators between HL and SE, so that we can develop strategies to improve them.

## Figures and Tables

**Table 1:** Characteristics of the Reviewed Studies

**Reference**	**Patient Characteristics**	**Study Method**	**Outcome Measures**	**Result**	**Conclusion**
Osborn et al. (2010)	383 White or African-American patients who were diagnosed with type 1 or type 2 diabetes mellitus, were age 18–85 years, and English speaking without any dementia, psychosis, or blindness	Research assistants collected data from participants in two primary care clinics and two diabetes specialty clinics located at three medical centers in the U.S.	HL: Rapid Estimate of Adult Literacy in Medicine; Numeracy: Wide Range Achievement Test, 3rd ed.; Diabetes SE: Perceived Diabetes Self-Management Scale	Both HL and numeracy were directly related to diabetes SE (*r* = 0.14, *p* < .01) (*r* = 0.17, *p* < .001).	There was a significant relationship between HL and SE and numeracy and SE
McCleary-Jones (2011)	50 African-American adults diagnosed with diabetes and who were able to speak and read English without cognitive deficit or known psychiatric illness	The participants were invited to complete the questionnaires in a community center in the U.S.	HL: Rapid Estimate of Adult Literacy in Medicine; SE: Diabetes SE scale	There is no correlation between HL and SE	HL was positively associated with diabetes knowledge, but not associated with SE
Ishikawa & Yano (2011)	157 Japanese patients with type 2 diabetes who were under continuous care by 1 of 4 attending physicians in the Department of Metabolic Disease	Participants were required to complete the baseline questionnaires initially and then complete the second questionnaires 4 weeks later in Japan	Patient communicative HL: Newly developed self-rated HL scale; SE in diabetes self-care: 4-item scale obtained from the self-care ability measure in the Diabetes Care Profile	Patient communicative HL was positively correlated with SE in diabetes self-care (*p* = .003, *p* = .052)	Communicative HL was positively associated with SE
White et al. (2013)	149 Hispanic patients who were age 18–85 years, able to speak Spanish fluently, had a corrected vision of ≤20/50, and did not have a history of psychosis or dementia	Trained research assistants interviewed participants during regular clinic hours for approximately 60 minutes at an adult community-based academic internal medicine clinic and two federally qualified health centers in the U.S.	HL: Short Test of Functional HL in Adults; SE: Perceived Diabetes Self-Management Scale	In unadjusted analyses, people with limited HL have shown greater SE for diabetes care than those with greater HL; after adjusted analyses, there is no relationship between SE and HL	HL was not correlated with SE
Inoue et al. (2013)	269 Japanese patients who were age 20–75 years, had been diagnosed with type 2 diabetes 1 year prior, and who presented to the clinic regularly were included. Patients age ≥75 years who had cognitive dysfunction or were unable to answer the questionnaires by themselves	Organizers provided the participants with questionnaires that they completed in health cooperative clinics in Japan	HL: Scales developed in Japan to assess functional, communicative, and critical HL of patients with diabetes; SE for diabetes management, fouritem scale of self-care ability in Diabetes Care Profile	Communicative and critical HL were positively associated with SE for diabetes management (β= 0.365, 0.369; *p*< .001)	Functional HL was not significantly associated with SE for diabetes management. On the other hand, both communicative and critical HL wer significantly associated with SE
Bohanny et al. (2013)	150 patients age >25 years with type 2 diabetes and able to speak and understand Marshallese were included; patients with impaired vision or psychiatric illness	Participants completed the study questionnaires in a public diabetes clinic in the U.S.	HL: Short Test of Functional HL in Adults; SE: Diabetes Management SE Scale	HL (*r*= 0.24, *p*= .003) was positively related to SE, and HL explained the highest variable (5%) of SE among HL, diabetes-related education (3.7%), and employment status (3.1%)	There was a strong positive relationship between HL and SE
Al Sayah et al. (2015)	343 patients with type 2 diabetes, age ≥18 years, and a clinic appointment between June 2010 and August 2010 were included; patients who did not speak English or were too ill or cognitively impaired	Eligible patients completed the study instruments in a private clinic room at two primary care clinics in the U.S.	HL: Three-item HL screening test; SE: Perceived Diabetes Self-Management Scale	HLQ1 was significantly associated with worse diabetes SE (*r*= 0.24, *p*< .0001). The total of the three HL items was weakly associated with diabetes SE (r = −.16, *p*< .005)	Inadequate HL was not linked with lower SE
Y. J. Lee et al. (2016)	295 Taiwanese patients with type 2 diabetes for >6 months, age 20–80 years, and the ability to read and communicate in Chinese were included	They completed the questionnaires at an endocrine outpatient clinic and at four local hospitals in Taiwan	HL: 14-item Japanese version diabetes HL scale, including functional, communicative, and critical HL; SE: 14-item Chinese version SE for diabetes management scale	Significant positive relationship was found between HL and SE (*p*< .001)	HL is directly significantly associated with SE among patients with type 2 diabetes
Reisi et al. (2016)	187 patients with type 2 diabetes without physical problems, mental disease, and cognitive dysfunction	Participants completed self-reported questionnaires in Iran	HL: Functional communicative and critical HL scale; SE: Diabetes Management SE Scale	Functional HL (*r*= 0.390, *p*< .010), communicative HL (*r*= 0.373, *p*< .010), and critical HL (*r*= 0.436, *p* < .010) were positively correlated with SE	Communicative, functional, and critical HL were significantly positively associated with SE
E.. H. Lee et al. (2016)	459 patients with type 2 diabetes were included; those with gestational diabetes	Participants completed a self-reported questionnaire in South Korea	HL: HL scale; SE: Diabetes Management SE Scale	Health literacy exerted direct effects on self-efficacy (ß = 0.450, *p*< .001)	HL was positively associated with SE
Zuercher et al. (2017)	381 patients with type 2 diabetes with a sufficient level of French language fluency without cognitive impairment or gestational diabetes	Participants completed a self-reported questionnaire in Switzerland	HL: A validated French version of a single-screening question assessing functional HL; SE: Stanford SE questionnaire	SE scores were associated with medium and poor functional HL (adjusted: ß = −.6, 95% CI [−.9, −.2], and ß = −1.8, 95% CI [−2.5, −1.2], respectively) after adjusting for potential confounders	People with poor functional HL had lower SE scores

Note. CI = confidence interval; HL= health literacy; HLQ1 = difficulty understanding written information (the first item on the HL screening test); SE = self-efficacy.
